# Differences in tibial subchondral bone structure evaluated using plain radiographs between knees with and without cartilage damage or bone marrow lesions - the Oulu Knee Osteoarthritis study

**DOI:** 10.1007/s00330-017-4826-8

**Published:** 2017-04-24

**Authors:** Jukka Hirvasniemi, Jérôme Thevenot, Ali Guermazi, Jana Podlipská, Frank W. Roemer, Miika T. Nieminen, Simo Saarakkala

**Affiliations:** 1 Research Unit of Medical Imaging, Physics and Technology, Faculty of Medicine, University of Oulu, POB 5000, FI-90014 Oulu, Finland; 2 Medical Research Center Oulu, Oulu University Hospital and University of Oulu, Oulu, Finland; 3 Infotech Oulu, University of Oulu, Oulu, Finland; 4 Quantitative Imaging Center, Department of Radiology, Boston University School of Medicine, Boston, MA USA; 5 Department of Radiology, University of Erlangen-Nuremberg, Erlangen, Germany; 6 Department of Diagnostic Radiology, Oulu University Hospital, Oulu, Finland

**Keywords:** Radiography, Osteoarthritis, Bone, Structural Analysis, Knee

## Abstract

**Objectives:**

To investigate whether subchondral bone structure from plain radiographs is different between subjects with and without articular cartilage damage or bone marrow lesions (BMLs).

**Methods:**

Radiography-based bone structure was assessed from 80 subjects with different stages of knee osteoarthritis using entropy of Laplacian-based image (E_Lap_) and local binary patterns (E_LBP_), homogeneity index of local angles (HI_Angles,mean_), and horizontal (FD_Hor_) and vertical fractal dimensions (FD_Ver_). Medial tibial articular cartilage damage and BMLs were scored using the magnetic resonance imaging osteoarthritis knee score. Level of statistical significance was set to *p* < 0.05.

**Results:**

Subjects with medial tibial cartilage damage had significantly higher FD_Ver_ and E_LBP_ as well as lower E_Lap_ and HI_Angles,mean_ in the medial tibial subchondral bone region than subjects without damage. FD_Hor_, FD_Ver_, and E_LBP_ were significantly higher, whereas E_Lap_ and HI_Angles,mean_ were lower in the medial trabecular bone region. Subjects with medial tibial BMLs had significantly higher FD_Ver_ and E_LBP_ as well as lower E_Lap_ and HI_Angles,mean_ in medial tibial subchondral bone. FD_Hor_, FD_Ver_, and E_LBP_ were higher, whereas E_Lap_ and HI_Angles,mean_ were lower in medial trabecular bone.

**Conclusions:**

Our results support the use of bone structural analysis from radiographs when examining subjects with osteoarthritis or at risk of having it.

***Key points*:**

• *Knee osteoarthritis causes changes in articular cartilage and subchondral bone*

• *Magnetic resonance imaging is a comprehensive imaging modality for knee osteoarthritis*

• *Radiography*-*based bone structure analysis can provide additional information of osteoarthritic subjects*

## Introduction

Bony changes, including osteophytes or subchondral cyst formation, are clearly seen on plain radiographs and are providing useful morphologic information in diseases affecting bone density and structure, such as osteoarthritis (OA) or osteoporosis. Although, the plain two-dimensional (2-D) radiograph is a projection (summation) through the actual three-dimensional (3-D) structure, bone density and bone structure as depicted by plain radiographs is significantly related with the actual 3-D structure of bone [1–5].

Diagnosis of OA is based on a subject’s history and symptoms, physical findings, and characteristic changes on plain radiographs. Typically, the severity of OA is evaluated from radiographs using the Kellgren–Lawrence (KL) grading scale, which is based on the visual evaluation of joint space narrowing, subchondral bone sclerosis, presence of osteophytes, and deformation of bone ends [6]. As ordinal grading using the KL scale gives only a summary score of overall disease severity with varying intra- and inter-rater reliability [7–9], development of quantitative and user-independent image analysis algorithms that exploit additional radiographic information is important to potentially enhance the clinical value of plain radiographs in OA diagnostics.

Joint space width is the most common parameter measured quantitatively from plain knee radiographs [10, 11]. Other parameters related to subchondral bone structure have also potential to be used as an additional measure in OA diagnostics and characterization with potential relevance for prediction of disease progression. Fractal analysis is the most popular method to assess bone structure from radiographs in OA research and a method called fractal signature analysis (FSA) has been shown to predict disease progression [12, 13]. Furthermore, it has been reported that bone structure assessed from plain radiographs using Laplacian-based method, local binary pattern (LBP)-based methods, and FSA is significantly related with the 3-D microstructure of bone [5]. Recently, subchondral and trabecular bone structures evaluated using LBP-based and Laplacian-based methods have shown to differ between subjects with different KL grades [14]. In that study, the KL grading and structure analysis of bone was made for the same images making the measurements dependent on each other to some extent, since features evaluated in the KL grading, for instance, bone sclerosis, affect the structural parameters as well. In order to study further the potential relevance of the radiography-based bone structural analysis methods, these should be compared with independent reference methods.

Magnetic resonance imaging (MRI) is considered the most comprehensive imaging modality for assessment of knee OA in a research context [15]. Semi-quantitative scoring systems that evaluate features related with or altered in the knee OA process have been developed and used for the assessment of structural deterioration of tissues within the knee joint [16]. Among many different features, MRI enables direct evaluation of cartilage damage and subchondral bone marrow lesions (BMLs) that are known to be related with OA incidence and progression [17–21]. However, the differences in subchondral bone structure from radiographs among subjects with and without morphological changes of articular cartilage or BMLs has not been thoroughly clarified yet. Given the fact that the local biomechanics are altered as a result of structural damage, radiographic bone structural changes are expected with prevalent cartilage damage and BMLs or both. Therefore, the aim of our study was to investigate whether subchondral bone structure from plain radiographs is different between subjects with and without articular cartilage damage and between subjects with and without BMLs focusing on the medial tibia.

## Methods

### Study subjects

This cross-sectional (level 3) study is part of the Oulu Knee OA (OKOA) study [22] and includes 80 subjects (49 women, 31 men) with different stages of symptomatic knee OA (KL grades from 0 to 4; Table 1). Written informed consent was obtained from each participant. The study was carried out in accordance with the Declaration of Helsinki and approved by the Ethical Committee of Northern Ostrobothnia Hospital District, Oulu University Hospital (number 108/2010).

**Table 1:** Description of the subjects (*n* = 80)

Parameter	Mean (standard deviation)	Min – max
Anthropometric variables		
Age (years)	60 (7.7)	34 – 70
Height (cm)	169 (7.6)	153 – 185
Weight (kg)	83 (14.3)	56 – 118
Body mass index (kg/m^2^)	29 (4.3)	21 – 42
Number of female subjects	49 (61.3%)	
KL grade distribution		
KL 0	2	
KL 1	21	
KL 2	20	
KL 3	20	
KL 4	17	

### Acquisition of radiographs

Weight-bearing posterior-anterior fixed-flexion radiographs of both knees with the X-ray beam angle at 10 degrees were obtained from all subjects. Since the original pixel size varied between images (mean: 138 μm, standard deviation: 20 μm, range: 100–168 μm), all images were resampled to the pixel size of 143 × 143 μm^2^ using bicubic interpolation to ensure comparability of the structural parameters, without producing as much artifacts as bilinear or nearest neighbor interpolation algorithms.

### Selection of regions of interests

**Fig. 1:**
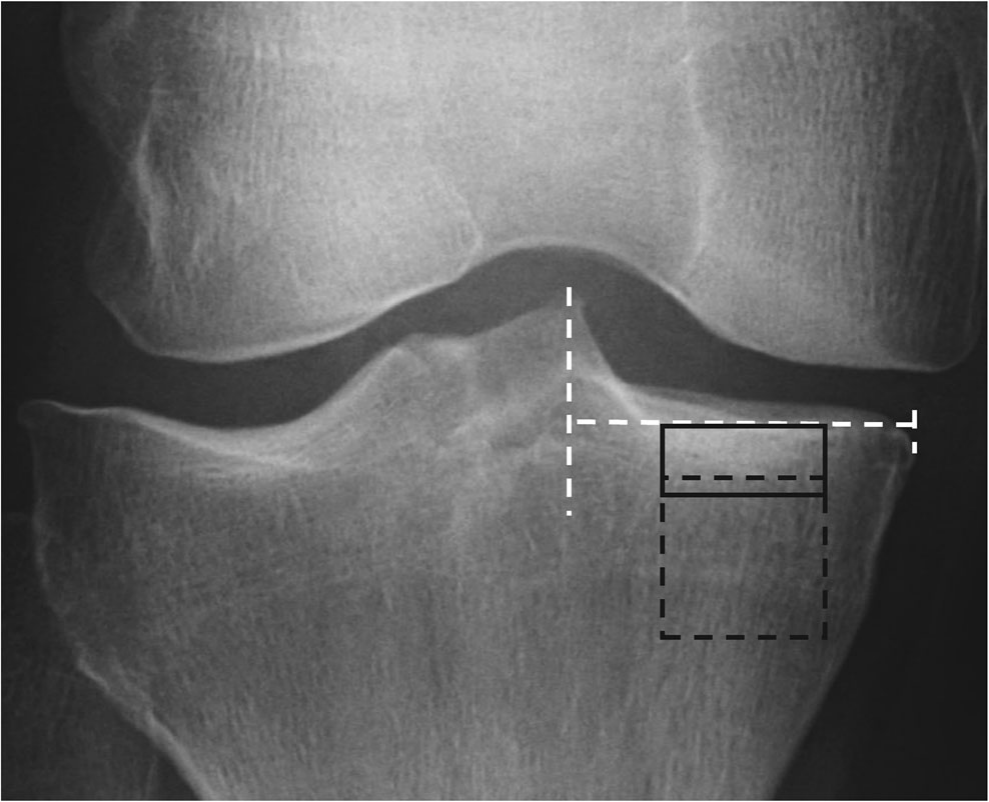
Location of regions of interest (ROIs). One ROI (*black rectangle* with *continuous line*) was placed in subchondral trabecular bone immediately below the cartilage-bone interface in the middle part of the medial tibial plateau. Another ROI (*black square* with *dashed line*) was placed immediately below the dense subchondral trabecular bone area. The purpose of the *white dashed lines* is to help place the ROIs in the middle of the tibial spine and outer border

Two rectangle-shaped regions of interests (ROIs) were defined (Fig. 1). The locations of the ROIs were based on previous literature [5, 14, 23, 24]. One ROI (size: 14 mm × 6 mm) was placed into the subchondral trabecular bone in the middle of the medial tibial plateau immediately below the cartilage-bone interface. This ROI is referred to as the subchondral bone ROI in this study, although different bone types are mixed in the ROI [25, 26]. Furthermore, another ROI (14 mm × 14 mm), referred to as trabecular bone ROI, was placed immediately below the dense subchondral trabecular bone. Trabecular bone ROIs were aligned horizontally with the subchondral bone ROIs. Anatomical landmarks for the ROIs were tibial spine, subchondral bone plate, and outer borders of the proximal tibia. A custom-made MATLAB software (version R2014b, The MathWorks, Inc., Natick, MA, USA) was used for the manual placement (JH) of the ROIs. Reproducibility of the texture parameters from the aforementioned locations has been shown to be high [14].

### Structural analysis of bone

Prior to the bone structural analysis, radiographs were median-filtered (3 × 3 pixels) to remove high-frequency noise and grayscale values of the image were expanded to full dynamic range. Bone structure was evaluated from the radiographs using Laplacian-based methods [5, 14, 27], LBP-based methods [5, 28] and FSA [5, 23, 29].

#### Laplacian-based analysis

The Laplacian-based method [5, 27] enhances the appearance of bone trabeculae and quantifies the variation in the grayscale values of the Laplacian-based image. Laplacians were calculated in the horizontal and vertical directions and summed into the one matrix. Subsequently, the unprocessed ROI was multiplied by the square root of the Laplacian matrix to enhance the bone, and grayscale values were expanded to the full dynamic range to obtain the final Laplacian-based image. To measure the randomness of the grayscale values in the Laplacian-based image, entropy of the image (E_Lap_) was calculated using the following equation:

1 $$ E=-{\displaystyle \sum_i{P}_i lo{g}_2{P}_i,} $$

where P_i_ contains the normalized count of the grayscale value *i* occurring in the image. If E_Lap_ = 0, all pixel values in the Laplacian-based image are the same, while higher values indicate higher variation in the pixel values of the image.

#### Local binary patterns (LBP)-based methods

LBP-based methods were used to measure the randomness of local patterns and the variations in the orientation of adjacent local patterns. First, the image was divided into bone and non-bone regions by determining a local threshold for every image pixel using the Otsu method [30] with a 9 × 9-pixel window size. Next, the LBP operator (eight-neighborhood on a circle with a radius of 1) was applied in the bone regions and the bone edges (i.e., non-bone regions next to the bone). The pixel was considered to be an edge pixel if at least one of the eight neighbors of the center pixel was a bone pixel. To reduce the number of irrelevant patterns, grouping of patterns was carried out by determining the main orientation and the number of valid neighbors (i.e., markers) for each pattern. The main orientation angle was calculated using principal component analysis. The angles (0°, 45°, 90°, and 135°) were calculated only for the patterns consisting of two to five consecutive markers; otherwise, the patterns were assigned as non-uniform.

To measure the randomness of the patterns occurring in the image, entropy of the grouped patterns (E_LBP_) was determined using the Eq. 1. If E_LBP_ = 0, there is only a single pattern occurring in the image.

The homogeneity index for the orientation of the valid patterns was derived from the co-occurrence matrix of the angles. The co-occurrence matrices were calculated in 0°, 45°, 90°, and 135° directions with one pixel distance. The non-uniform and non-bone areas were excluded from the co-occurrence matrices. To take into account the orientation of bone trabeculae in the analysis, co-occurrence matrices of 0° and 135° directions were combined together as well as 45° and 90° directions to calculate the homogeneity index perpendicularly to the bone trabeculae (HI_Angles,Perp_) and along the trabeculae (HI_Angles,Paral_), respectively. Furthermore, the mean homogeneity index (HI_Angles,mean_) was calculated from the co-occurrence matrix as the sum of the four possible directions. The interpretation of the HI_Angles_ parameters used in this study is the following: If all adjacent patterns have similar orientation, HI_Angles_ is equal to one, while a large variation in the orientation of local patterns results in a low HI_Angles_ value.

#### Fractal signature analysis (FSA)

To estimate fractal dimension, related to roughness and complexity of an image, the FSA method was used [23, 29]. In brief, the image was dilated and eroded in horizontal and vertical directions with a rod-shaped one-pixel-wide structuring element and the volume, *V*, between dilated and eroded images was calculated. Calculations were repeated by varying the element length *r* from two to four pixels. The surface area, *A*(*r*), was calculated using the Eq. 2:

2 $$ A(r)=\frac{V(r)- V\left( r-1\right)}{2}, $$

Subsequently, a log-log plot was constructed by plotting log of *A*(*r*) against log of *r*. Finally, the fractal dimension was estimated using a regression line to points between 2 and 4. We restricted our analyses only to the small-scale fractal dimensions in order to keep the results compact. When the structuring element is pointing in the horizontal direction, fractal dimension of vertical structures (FD_Ver_) is produced and vice versa. High fractal dimension values are associated with high complexity of the image, whereas low complexity results in low fractal dimension values.

### Magnetic resonance imaging

**Fig. 2:**
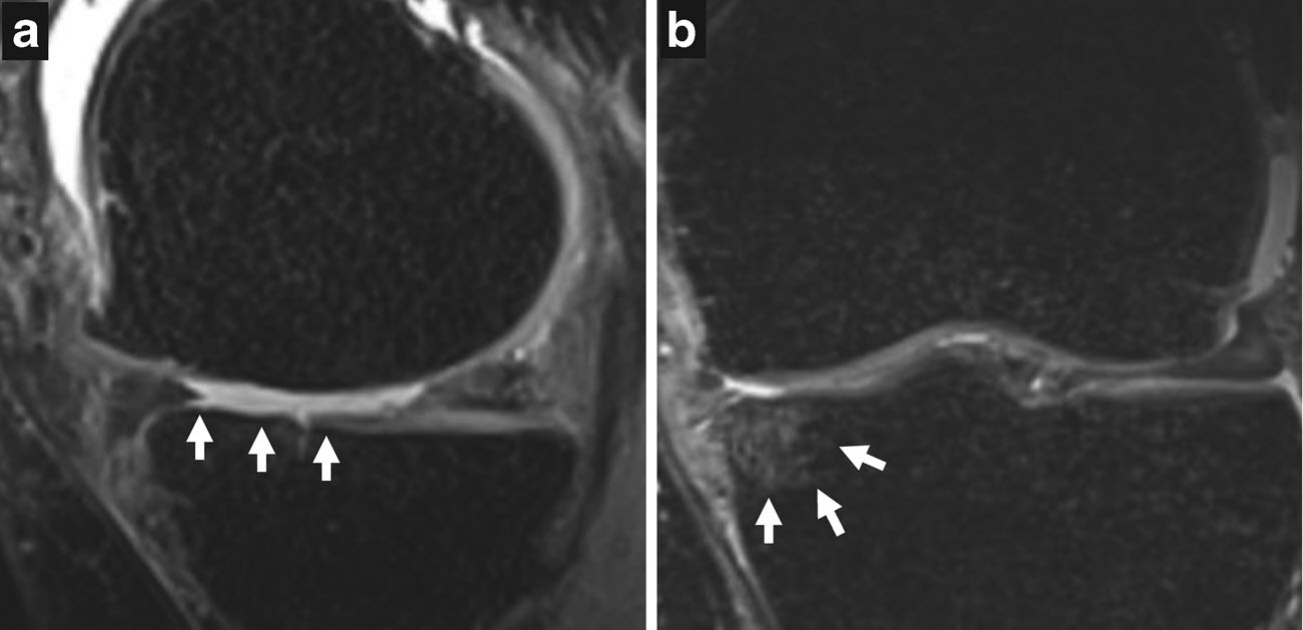
Example MR images obtained using proton density-weighted SPACE fat-suppressed turbo spin-echo sequence. A) Grade 3 for the size of cartilage loss in anterior and grade 2 cartilage loss in central medial tibia (*arrows*). B) Grade 2 for the size of bone marrow lesion in medial central tibia (*arrows*)

Within 31– 227 (mean: 125, standard deviation: 49) days from the date of radiography, all subjects were scanned with a 3-Tesla (T) MRI scanner (Siemens Skyra, Siemens Healthcare, Erlangen, Germany) using a 15-channel transmit/receive knee coil. The following sequences were carried out: sagittal T2-weighted dual-echo steady-state [repetition time (TR): 14.1 ms, echo time (TE): 5 ms, echo train length (ETL): 2, field of view (FOV): 150 × 150 mm^2^, acquisition matrix: 256 × 256, slice thickness: 0.6 mm], 3-D sagittal proton-density (PD)-weighted SPACE fat-suppressed turbo spin-echo (TSE; TR: 1200 ms, TE: 26 ms, ETL: 49, FOV: 160 × 160 mm^2^, acquisition matrix: 256 × 256, slice thickness: 0.6 mm), coronal PD-weighted TSE (TR: 2800 ms, TE: 33 ms, ETL: 4, FOV: 140 × 140 mm^2^, acquisition matrix: 384 × 384, slice thickness: 3 mm) and coronal T1-weighted TSE (TR: 650 ms, TE: 18 ms, ETL: 2, FOV: 130 × 130 mm^2^, acquisition matrix: 320 × 320, slice thickness: 3 mm). A musculoskeletal radiologist (AG, 15 years of experience in semi-quantitative MRI analysis of knee OA) scored the medial articular cartilage damage and BMLs using the MRI OA knee score (MOAKS) [16]. A subject was included in the medial tibial cartilage damage group if he/she had any cartilage loss [defined as MOAKS grades 1–3 for the size of any cartilage loss (including partial and full-thickness loss)] in the medial anterior, central, or posterior tibia (Fig. 2A). Similarly, a subject was included in the medial tibial BML group if he/she had any BML (MOAKS grades 1–3 for the size of BML by volume; including ill-defined lesions, bone marrow edema and subchondral cysts) in the medial anterior, central, or posterior part of tibia (Fig. 2B).

### Statistical analyses

Analysis of covariance was used to compare the bone structure between groups with and without cartilage damage or BMLs. Bone structural parameters were adjusted with gender, age and body mass index. Unadjusted mean values (standard deviations) are shown in the result tables. Mean KL grade and its standard deviation are reported in Tables 2 and 3 for clarity although the KL grade is an ordinal scale variable. Difference in the KL grade between groups was tested using the Mann–Whitney U test. Statistical analyses were conducted using IBM SPSS Statistics for Windows (Version 22.0, IBM Corp., Armonk, NY, USA).

**Table 2:** Mean (standard deviation) of anthropometric variables, KL grade, original pixel size of the image and bone structural parameters among subjects without and with medial tibial cartilage damage

Parameter	No lesion (*n* = 31)	Medial tibial cartilage damage (*n* = 49)	*p* value	Adjusted *p* value*
Age (years)	57.5 (9.0)	61.5 (6.5)	0.039	
Height (cm)	167.7 (7.0)	169.8 (8.0)	0.240	
Weight (kg)	82.0 (16.8)	83.7 (12.6)	0.603	
Body mass index (kg/m^2^)	29.0 (4.7)	29.1 (4.1)	0.917	
KL grade	1.61 (0.99)	2.84 (1.01)	<0.001^a^	
Original pixel size (μm)	136 (20)	140 (20)	0.398	
Medial subchondral bone				
FD_Hor_	2.51 (0.14)	2.55 (0.13)	0.246	0.078
FD_Ver_	2.75 (0.08)	2.84 (0.10)	<0.001	<0.001
E_Lap_	6.99 (0.17)	6.88 (0.16)	0.008	0.002
E_LBP_	3.72 (0.05)	3.74 (0.04)	0.025	0.004
HI_Angles,mean_	0.69 (0.01)	0.68 (0.01)	0.010	0.006
HI_Angles,Perp_	0.68 (0.01)	0.67 (0.01)	0.001	0.002
HI_Angles,Paral_	0.70 (0.01)	0.70 (0.01)	0.148	0.047
Medial trabecular bone				
FD_Hor_	2.60 (0.09)	2.65 (0.08)	0.013	<0.001
FD_Ver_	2.82 (0.10)	2.88 (0.10)	0.009	0.001
E_Lap_	6.91 (0.13)	6.82 (0.12)	0.003	<0.001
E_LBP_	3.62 (0.08)	3.66 (0.06)	0.034	0.003
HI_Angles,mean_	0.71 (0.01)	0.71 (0.01)	0.011	0.001
HI_Angles,Perp_	0.69 (0.01)	0.68 (0.01)	0.021	0.002
HI_Angles,Paral_	0.74 (0.02)	0.73 (0.01)	0.015	0.003

^*^Adjusted for gender, age and body mass index. ^a^ Tested with Mann–Whitney U test. FD = fractal dimension of horizontal (Hor) or vertical (Ver) structures, E_Lap_ = entropy of Laplacian-based image, E_LBP_ = entropy of grouped local binary patterns, HI_Angles_ = homogeneity index (HI) for orientation of local patterns, HI_Angles,Perp_ = HI perpendicularly to the bone trabeculae, HI_Angles,Paral_ = HI along the trabeculae

**Table 3:** Mean (standard deviation) of anthropometric variables, KL grade, original pixel size of the image and bone structural parameters among subjects without and with medial tibial BML

Parameter	No medial tibial BML (*n* = 51)	Medial tibial BML (*n* = 29)	*p* value	Adjusted *p* value*
Age (years)	58.4 (8.5)	62.7 (5.4)	0.008	
Height (cm)	168.6 (7.7)	169.6 (7.7)	0.575	
Weight (kg)	81.2 (15.8)	86.3 (10.5)	0.121	
Body mass index (kg/m^2^)	28.5 (4.7)	30.1 (3.2)	0.079	
KL grade	1.78 (0.97)	3.38 (0.68)	<0.001^a^	
Original pixel size (μm)	137 (20)	140 (20)	0.570	
Medial subchondral bone				
FD_Hor_	2.52 (0.13)	2.56 (0.12)	0.163	0.052
FD_Ver_	2.79 (0.10)	2.84 (0.09)	0.019	0.007
E_Lap_	6.95 (0.16)	6.88 (0.18)	0.046	0.020
E_LBP_	3.73 (0.04)	3.74 (0.03)	0.043	0.024
HI_Angles,mean_	0.69 (0.01)	0.68 (0.01)	0.002	0.001
HI_Angles,Perp_	0.68 (0.01)	0.67 (0.01)	0.001	0.001
HI_Angles,Paral_	0.70 (0.01)	0.70 (0.01)	0.019	0.011
Medial trabecular bone				
FD_Hor_	2.61 (0.09)	2.65 (0.08)	0.030	0.007
FD_Ver_	2.84 (0.11)	2.89 (0.09)	0.036	0.001
E_Lap_	6.88 (0.13)	6.80 (0.14)	0.007	0.003
E_LBP_	3.63 (0.07)	3.67 (0.07)	0.017	0.011
HI_Angles,mean_	0.71 (0.01)	0.70 (0.01)	0.004	0.002
HI_Angles,Perp_	0.69 (0.01)	0.68 (0.01)	0.007	0.003
HI_Angles,Paral_	0.73 (0.01)	0.73 (0.01)	0.005	0.006

^*^Adjusted for gender, age and body mass index. ^a^ Tested with Mann–Whitney U test. FD = fractal dimension of horizontal (Hor) or vertical (Ver) structures, E_Lap_ = entropy of Laplacian-based image, E_LBP_ = entropy of grouped local binary patterns, HI_Angles_ = homogeneity index (HI) for orientation of local patterns, HI_Angles,Perp_ = HI perpendicularly to the bone trabeculae, HI_Angles,Paral_ = HI along the trabeculae

## Results

**Fig. 3:**
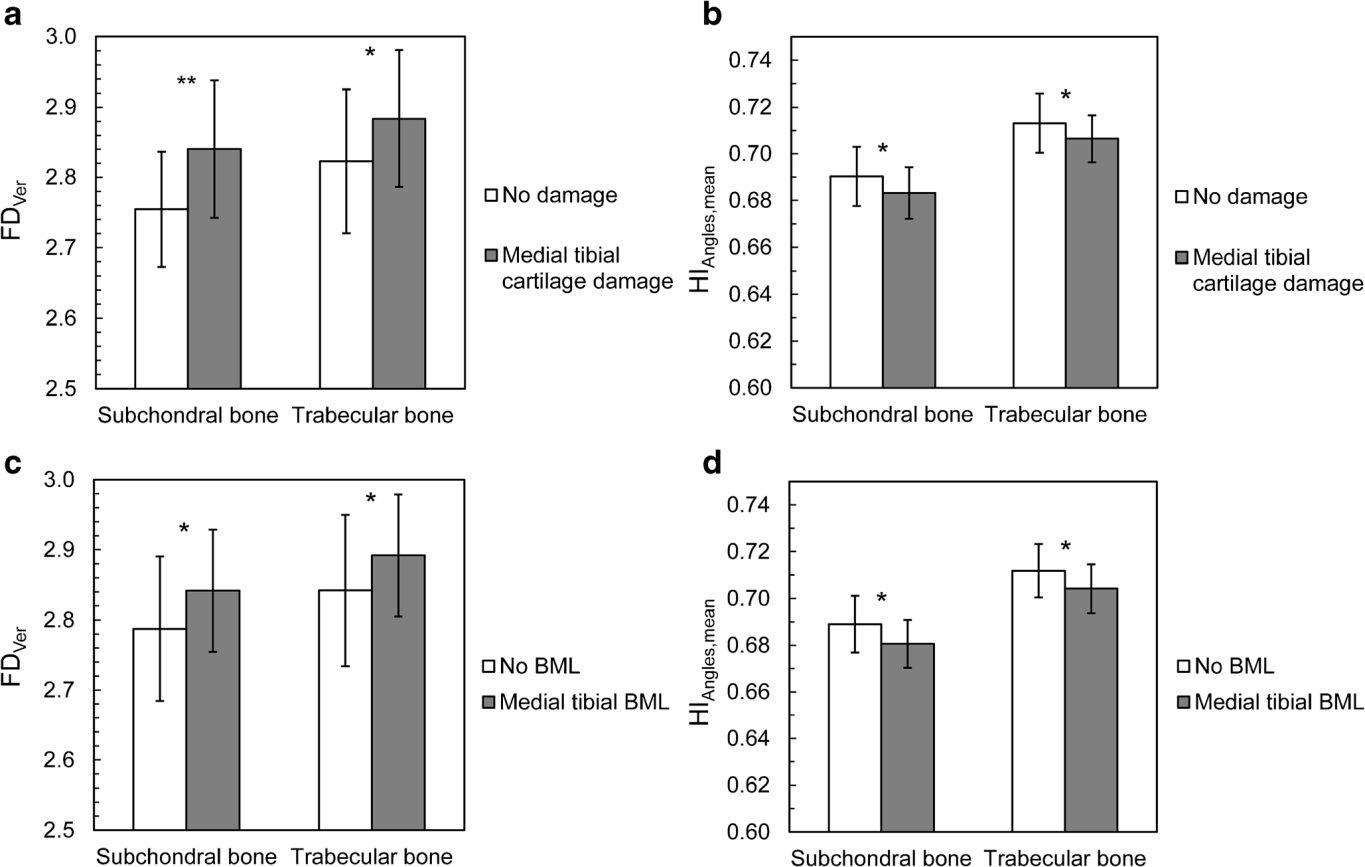
Statistically significant differences in bone structural parameters (A) FD_Ver_ and (B) HI_Angles,mean_ between subjects with and without medial tibial cartilage damage, and (C) FD_Ver_ and (D) HI_Angles,mean_ between subjects with and without medial tibial bone marrow lesions (BMLs). *p < 0.05, **p < 0.001

Subjects with medial tibial cartilage damage had significantly different bone structure in medial tibial subchondral and trabecular bone ROIs (Table 2). In the medial subchondral bone region, FD_Ver_ (Fig. 3A) and E_LBP_ were higher, whereas E_Lap_, HI_Angles,mean_ (Fig. 3B), HI_Angles,Perp_, and HI_Angles,Paral_ were lower among subjects with medial tibial cartilage damage (Table 2). In the medial trabecular bone region, FD_Hor_, FD_Ver_ (Fig. 3A), and E_LBP_ were higher, whereas E_Lap_, HI_Angles,mean_ (Fig. 3B), HI_Angles,Perp_, and HI_Angles,Paral_ were lower among subjects with medial tibial cartilage damage (Table 2).

Subjects with medial tibial BMLs had also significantly different bone structure in medial tibial subchondral and trabecular bone ROIs (Table 3). In the medial subchondral bone region, FD_Ver_ (Fig. 3C) and E_LBP_ were higher, whereas E_Lap_, HI_Angles,mean_ (Fig. 3D), HI_Angles,Perp_, and HI_Angles,Paral_ were lower among subjects with medial tibial BMLs (Table 3). In the medial trabecular bone region, FD_Hor_, FD_Ver_ (Fig. 3C), and E_LBP_ were higher, whereas E_Lap_, HI_Angles,mean_ (Fig. 3D), HI_Angles,Perp_, and HI_Angles,Paral_ were lower among subjects with medial tibial BMLs (Table 3).

When tibial cartilage damage analyses were adjusted for medial tibial BMLs along with gender, age, and body mass index, FD_Ver_ was higher (*p* = 0.003) and E_Lap_ was lower (*p* = 0.023) among subjects with medial cartilage damage in the medial subchondral bone region. In the medial trabecular bone region, FD_Hor_ (*p* = 0.016) and FD_Ver_ (*p* = 0.042) were higher, whereas E_Lap_ was lower (*p* = 0.023). When BML analyses were adjusted for medial cartilage damage along with gender, age, and body mass index, significantly higher HI_Angles,mean_ values (*p* = 0.036) between the control and medial tibial BML groups was detected.

When tibial cartilage damage analyses were adjusted for KL grade, gender, age, and body mass index, FD_Ver_ was higher (*p* = 0.001) and E_Lap_ was lower (*p* = 0.008) among subjects with medial cartilage damage in the medial subchondral bone region. In the medial trabecular bone region, FD_Hor_ (*p* = 0.020), FD_Ver_ (*p* = 0.005) and E_LBP_ (*p* = 0.030) were higher, whereas E_Lap_ was lower (*p* = 0.009). When BML analyses were adjusted for the KL grade gender, age, and body mass index, only HI_Angles,Perp_ was significantly lower (*p* = 0.041) in medial subchondral bone ROIs, and FD_Ver_ was significantly higher (*p* = 0.006) in medial trabecular bone ROIs among subjects with medial tibial BMLs.

## Discussion

Our results demonstrate that medial tibial subchondral and trabecular bone structures from 2-D plain radiographs were different between subjects with and without medial tibial articular cartilage damage and between subjects with and without medial tibial BMLs.

The finding that trabecular bone structure from radiographs is different between subjects with and without cartilage damage is in line with an earlier study [31]. In that study, the fractal dimension of horizontal structures in the lateral compartment and fractal dimension of vertical structures in both medial and lateral compartments were significantly higher among subjects with cartilage defects (in medial, lateral, or both compartments) compared to subjects without cartilage defects [31]. As a complement to that study, the subchondral bone area was also analyzed in the current study. Furthermore, we used novel LBP- and Laplacian-based algorithms along with FSA to assess bone structure. As a novel finding, we were able to confirm that, in addition to trabecular bone structure, the structure of subchondral bone assessed from 2-D radiographs is also different between subjects with and without medial cartilage damage. Consequently, the assessment of the subchondral bone area immediately under the cartilage-bone interface should be considered in future studies using plain radiographs as that area has an important role in OA pathology [25, 26].

Bone structural parameters in both subchondral and trabecular bone ROIs were different between subjects with and without BMLs in medial tibia. The higher fractal dimension and E_LBP_ and lower HI_Angles_ values suggest that the bone structure is more disoriented among subjects with medial tibial BMLs. In a previous MRI study, subjects with BMLs had higher apparent bone volume fraction (calculated from MRI data), higher trabecular thickness and number, and lower trabecular separation [32]. The current results are in line with that study since, for example, higher FD_Ver_ and E_LBP_ and lower HI_Angles,mean_ values are known to be related with lower trabecular separation values [5]. It is known that BMLs are more common among subjects with advanced OA and this might explain partly the observed differences in the calculated bone structural parameters. It should also be noted that all subjects with BMLs had concurrent cartilage damage which further limits our BML-related analyses.

In this study, we showed that FSA, Laplacian-based and LBP-based methods are able to detect differences between subjects with and without cartilage damage or BMLs. One explanation for the differences in structural parameters of bone is that subchondral and trabecular bone in the proximal tibia is not as well-organized among subjects with cartilage damage or BMLs. This explanation is supported by the previous studies which have proposed that the trabecular bone is more disorganized and the orientation of trabecular bone is shifted, probably due to abnormal loading, in OA [33–35]. With the FSA method, fractal dimensions of vertical and horizontal structures are typically reported [12, 29, 36–38]. However, bone trabeculae are not perfectly oriented vertically in the proximal tibia. To account for this, HI_Angles_ was calculated approximately perpendicularly and parallel to bone trabeculae. It should be noted that due to differences in calculation of fractal dimension and HI_Angles_ values, FD_Ver_ and HI_Angles,Perp_ (and FD_Hor_ and HI_Angles,Paral_) are related to each other, although these variables are not directly measuring the same phenomenon.

One limitation of our study is the difference in imaging conditions of radiographs. The conditions varied because the study subjects were imaged at different institutions on different imaging systems. Since texture analysis methods used in this study are affected by pixel size, the comparability of the calculated structural parameters between different images was ensured by resampling the pixel size of all images to 143 × 143 μm^2^. Furthermore, texture analysis methods are not very sensitive for the differences in imaging conditions (e.g., tube voltage and current), as the methods do not usually evaluate plain grayscale values directly. Another limitation is that many of the subjects had multiple concurrent tissue changes in MOAKS. However, due to limited sample size and restricted knowledge on the most important factors for the bone structure appearance as determined from radiographs, we decided not to adjust bone analyses with MOAKS features. Furthermore, in the OKOA data, radiographs were available only for symptomatic subjects and, thus, our “control” groups might have had some OA-related changes. On the other hand, it is likely that the differences would have been even higher if non-symptomatic true controls were used. Finally, since our study design was cross-sectional, we could not study the causality of the tissue changes; i.e., it is not possible to determine whether the cartilage damage or BML induces the alteration in the subchondral bone structure or vice versa, for example. The longitudinal relevance of the subchondral bone structure analysis from plain radiographs warrants further exploration.

Plain radiography is a cheap, fast, and widely available imaging method suitable for imaging of large subject cohorts. Analysis of subchondral bone structure can be performed relatively fast using the presented methods. The only manual user input needed is to place the ROIs in the correct pre-defined location, which takes about one minute depending on the experience of the user. The remaining analyses are performed by the software algorithm. Semi-automated and automated methods have also been developed for the ROI placement [39–41]. In this study, we confirmed that subchondral and trabecular bone structure from radiographs is different among subjects with cartilage damage or BMLs, which both are significantly related with OA incidence and progression [17–21]. Therefore, our results suggest that structural analysis of bone from radiographs can be used as a supplementary tool when evaluating subjects with OA or at risk of having it.

## Abbreviations

BML: bone marrow lesion
E_Lap_: entropy of Laplacian-based image
E_LBP_: entropy of grouped Local Binary Patterns
FD_Hor_: fractal dimension of horizontal structures
FD_Ver_: fractal dimension of vertical structures
FSA: fractal signature analysis
HI: homogeneity index
HI_Angles_: HI for orientation of local patterns
HI_Angles,Perp_: HI perpendicularly to the bone trabeculae
HI_Angles,Paral_: HI along the trabeculae
KL: Kellgren–Lawrence
LBP: local binary patterns
MOAKS: MRI OA knee score
MRI: magnetic resonance imaging
OA: osteoarthritis
PD: proton density
TSE: turbo spin-echo
ROI: region of interest
2-D: two-dimensional
3-D: three-dimensional
